# Affinity Peptide-Based Circularly Permuted Fluorescent Protein Biosensors for Non-Small Cell Lung Cancer Diagnosis

**DOI:** 10.3390/s24247899

**Published:** 2024-12-11

**Authors:** Dengyue Xu, Qingyun Jiang, Zhi Li, Angyang Shang, Jiaqi Liu, Chengyu Xue, Shuai Shao, Hangyu Zhang, Hong Yuan, Bin Wu, Bo Liu

**Affiliations:** 1 Central Hospital of Dalian University of Technology, Dalian 116021, China; xudengyue@dlut.edu.cn (D.X.); jiangqingyun@mail.dlut.edu.cn (Q.J.); postlizhi@126.com (Z.L.); anyashang@mail.dlut.edu.cn (A.S.); ljiaqi@mail.dlut.edu.cn (J.L.); ltts23455@mail.dlut.edu.cn (C.X.); shaos@dlut.edu.cn (S.S.); hangyuz@dlut.edu.cn (H.Z.); yuanhonglab@163.com (H.Y.); 2 Liaoning Key Lab of Integrated Circuit and Biomedical Electronic System, Faculty of Medicine, Dalian University of Technology, Dalian 116024, China; 3 China Certification and Inspection Group Liaoning Co., Ltd., Dalian 116039, China

**Keywords:** non-small cell lung cancer, carcinoembryonic antigen, squamous cell carcinoma antigen, cytokeratin fragment 19, circularly permuted fluorescent protein, phage display

## Abstract

Non-small cell lung cancer (NSCLC) is the predominant form of lung cancer and poses a significant public health challenge. Early detection is crucial for improving patient outcomes, with serum biomarkers such as carcinoembryonic antigen (CEA), squamous cell carcinoma antigen (SCCAg), and cytokeratin fragment 19 (CYFRA 21-1) playing a critical role in early screening and pathological classification of NSCLC. However, due to being mainly based on corresponding antibody binding reactions, existing detection technologies for these serum biomarkers have shortcomings such as complex operations, high false positive rates, and high costs. This study aimed to develop new methods for detecting CEA, SCCAg, and CYFRA 21-1 to assist in the diagnosis of NSCLC. Affinity peptides of CEA, SCCAg, and CYFRA 21-1, respectively, were screened by phage display technology, and the peptides’ binding affinities were determined by enzyme-linked immunosorbent assay and biolayer interferometry. Peptides with high affinity were then integrated as binding domains into biosensors by fusing them with circularly permuted fluorescent proteins (cpFPs) through genetic coding. The resulting biosensors, C4 biosensor for CEA, S1 biosensor for SCCAg, and Y3 biosensor for CYFRA 21-1, demonstrated robust sensitivity and specificity even at concentrations as low as 1 ng/mL for their respective tumor markers. When applied to clinical samples and recalibrated for the upper limit of normal concentrations, the biosensors exhibited enhanced sensitivity and specificity for NSCLC diagnosis. This study introduced innovative biosensors for the detection of CEA, SCCAg, and CYFRA 21-1, providing a highly sensitive, specific, rapid, and cost-effective diagnostic alternative that could significantly improve NSCLC screening rates.

## 1. Introduction

Lung cancer stands as one of the most prevalent and deadly malignancies, posing a significant threat to global public health [1]. According to projections, the cancer burden is expected to double by 2050, with lung cancer leading the list of concerns [2]. Within this spectrum, non-small cell lung cancer (NSCLC) is the predominant subtype, accounting for an alarming 80–85% of all lung cancer cases [3]. The subtle clinical symptoms of early-stage lung cancer often lead to a delayed diagnosis, with the majority of patients only seeking medical attention when the disease has progressed to advanced stages. This reality underscores the urgent need for effective early screening methods for NSCLC, a challenge that remains at the forefront of cancer research [4]. Histopathological examination, while considered the gold standard of lung cancer diagnosis, is limited by its invasive nature, high cost, time-consuming procedures, and the difficulty in obtaining tumor samples [5]. In contrast, serum tumor markers (STMs), a class of protein-based circulating molecules, offer a non-invasive and repeatable approach to detection. STMs are produced by the body or tumor in response to cancer or certain benign conditions, making them invaluable tools in the clinical auxiliary diagnosis of NSCLC [6]. Extensive research has highlighted the clinical significance of STMs such as carcinoembryonic antigen (CEA), squamous cell carcinoma antigen (SCCAg), and cytokeratin fragment 19 (CYFRA 21-1) in the context of NSCLC [7]. The detection of these markers not only aids in the diagnosis of NSCLC but also plays a crucial role in early screening and pathological classification of lung cancer. However, current detection methodologies for STMs, such as enzyme-linked immunosorbent assay (ELISA), electrochemiluminescence, and radioimmunoassay [8], are hindered by the need for substantial operational equipment, complex experimental procedures, and high cost, limiting their widespread application. There is, therefore, an urgent need to develop a novel detection approach for NSCLC that is operationally straightforward, cost-effective, rapid, and delivers accurate results.

Genetically encoded fluorescent biosensors have transformed the visualization and quantification of enzyme activity, protein conformational changes, specific molecular concentration, and biophysical parameters within living cells, tissues, and even entire organisms [9]. Among these biosensors, circularly permuted fluorescent protein (cpFP) stands out due to its effective coupling of the chromophore with the receptive domain, which significantly enhances the dynamic response range of the fluorescent protein [10]. cpFP can provide a sufficient signal-to-noise ratio for measurement, even if the binding domain protein undergoes conformational rearrangement. Its low molecular weight facilitates the optimization of protein expression rate and enhances its subcellular localization, which is pivotal for intracellular studies [11]. Moreover, cpFP occupies a narrow spectral linewidth, a significant advantage for multi-parameter imaging, allowing for the simultaneous monitoring of multiple biochemical events within a single living system [12]. Based on the above merits, cpFP has emerged as a prominent tool in the field of biosensors and bio-imaging, offering a versatile platform for research and diagnostics.

Peptides are typically short linear chains consisting of fewer than 50 amino acid residues without complex tertiary structures, and their functionality is primarily dictated by their sequence of amino acids [13]. In comparison to antibodies or other proteins, peptides exhibit a smaller molecular size, which endows them with superior tissue penetration capabilities [14]. Moreover, peptides have a significantly reduced likelihood of being recognized by immune cells, thereby reducing peptide-specific antibodies or eliciting peptide-specific T cell responses. This attribute confers upon peptides a lower immunogenicity and enhanced biocompatibility [15]. Phage display technology, recognized with the Nobel Prize in Chemistry in 2018, is a powerful approach for the screening of affinity peptides with high affinity and selectivity for target proteins [10]. These affinity peptides identified through this method can be engineered to conjugate with imaging indicators or therapeutic drugs, thereby forming specific biosensors or targeted drugs [16].

**Figure 1:**
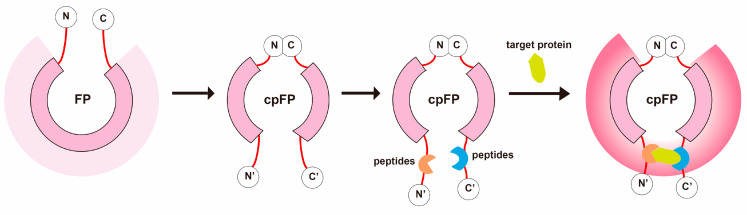
Construct Design of the Circularly Permuted Fluorescent Protein (cpFP) Biosensor. This schematic illustrates the assembly of the cpFP biosensor. The native N- and C-termini of the cpFP are joined, creating new termini at alternative tolerance sites. The affinity peptides, identified through screening, are then attached to these new termini, serving as the binding domain proteins. Upon binding to the target protein, the microenvironment surrounding the chromophore of the rearranged fluorescent protein alters, leading to a significant fluorescence response.

To establish an alternative detection method based on antibody binding reaction, this research combines the advantages of affinity peptides and cpFP to develop a set of novel cpFP biosensors for detecting STMs of NSCLC (Figure 1). Affinity peptides were obtained through phage display, ELISA, and biolayer interferometry (BLI) using CAE, SCCAg, and CYFRA 21-1 as target proteins, and cpFP biosensors with corresponding affinity peptides as binding domains were constructed through genetic engineering. It provided powerful tools for the highly sensitive, specific, rapid, and cost-effective detection of CAE, SCCAg, and CYFRA 21-1, paving the way for early NSCLC screening and diagnosis and ultimately aiming to significantly improve the overall prognosis of NSCLC patients.

## 2. Materials and Methods

### 2.1. Phage Display

The Ph.D.-12 Phage Display Peptide Library Kit (E8110SC, New England BioLabs, Ipswich, MA, USA) was employed for screening in 96-well plates. Plates were precoated with 100 μg/mL target protein in bicarbonate buffer (pH 8.6) at 4 °C overnight to facilitate protein adsorption. After blocking with bovine serum albumin (BSA), the plates were incubated with the phage library for 60 min at room temperature on a shaker to allow phage binding to the target proteins. Non-specifically bound phages were removed by washing with TBST buffer (10 washes). Bound phages were eluted using a Glycine-BSA buffer (0.2 M glycine-HCl, 1 mg/mL BSA, pH 2.2) and amplified. This three-round panning process enriched for high-affinity phages. After the final round, 30 blue colonies from Luria-Bertani (LB) plates were selected for sequencing. Dideoxy sequencing of phage DNA determined the peptide sequences responsible for target binding [17].

### 2.2. ELISA

Based on sequencing outcomes, the affinity of amplified monoclonal phages for the target marker was assessed using ELISA. Proteins (100 μg/mL) were coated onto 96-well plates (200 μL/well) and blocked with 300 μL of BSA. Phage particles (10^10^, 100 μL) were added per well and incubated at 37 °C for 1 h. Following six washes with TBST, the plates were incubated with 100 μL of a 1:5000 dilution of horseradish peroxidase-conjugated anti-M13 antibody (11073, SinoBiological, Beijing, China) at room temperature for 1 h. After further washing, the reaction was developed with an o-phenylenediamine (OPD, P6662, Sigma-Aldrich, St. Louis, MO, USA) substrate in the dark, and absorbance was measured at 492 nm.

### 2.3. Peptide Synthesis

High-affinity peptides identified through prior analyses were synthesized using the standard solid-phase fluorenylmethoxycarbonyl (Fmoc) method by Synpeptide Co., Ltd. (Shanghai, China). The synthesized peptides were purified by high-performance liquid chromatography (HPLC) to achieve a purity of at least 95% and were characterized by mass spectrometry (MS).

### 2.4. BLI

The binding affinities of the peptides to their respective markers were assessed using an Octet RH96 instrument (Sartorius AG, Goettingen, Germany) with software version 12.2.1.18. Markers CEA, SCCAg, and CYFRA 21-1 were immobilized on amine reactive second-generation biosensors (AR2G, Sartorius AG) via amine coupling, with ethanolamine used to quench unreacted sites. Peptides were prepared in a range of concentrations. Experiments were conducted at room temperature and involved three phases: baseline (60 s), association (90 s), and dissociation (180 s). Data analysis was performed using Octet Data Analysis software version 11.0 to calculate the equilibrium dissociation constant (K_D_) as dissociation constant (kd)/association constant (ka). Results were plotted using WPS Office Excel (version 12.1.0.16929).

### 2.5. Plasmid Construction

**Figure 2:**
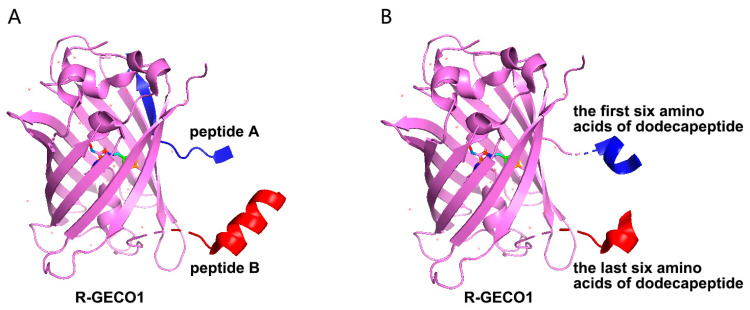
The strategy for the construction of the cpFP biosensor. (**A**): Using R-GECO1 as a template, the cpFP biosensor was constructed with two selected dodecapeptides as the N-terminus and C-terminus, respectively. (**B**): Using R-GECO1 as a template, the cpFP biosensor was constructed with two hexapeptides separated from the selected dodecapeptide as the N-terminus and C-terminus.

The circularly permuted red fluorescent protein R-GECO1 was employed as a template for biosensor engineering [18]. Two high-affinity peptides per marker were identified via LBI detection. Plasmids were constructed using two strategies: one with peptides as N- and C-termini of cpFP and another with dodecapeptides split into hexapeptides inserted at the termini. Three sequences per marker were generated and cloned into the BamHI and EcoRI sites of pRSETB with a 6× His tag (Figure 2).

### 2.6. Protein Expression and Purification

Plasmids were transformed into *E. coli* BL21 (DE3) cells for protein expression. Cultures were grown in LB medium with ampicillin at 37 °C for 6 h, then induced with IPTG at 17 °C for 4 h. Cell pellets were lysed in buffer containing 10 mM of imidazole, 50 mM of NaH2PO4, and 300 mM of NaCl (pH 7.4), centrifuged at 4 °C (10,000× *g*, 20 min), and the supernatant was collected. Purification was performed on an AKTA instrument, and proteins were dialyzed in PBS at 4 °C before being packaged and stored protected from light at −80 °C.

### 2.7. Fluorescence Detection

A microplate reader was used to measure fluorescence emissions at 600 nm with the excitation at 490 nm. The sensitivity, specificity, and standard curves of the biosensors were assessed using a reaction system comprising 180 μL of biosensor solution and 20 μL of the test substance. Neuron-specific enolase (NSE) and pro-gastrin-releasing peptide (proGRP), crucial biomarkers for diagnosing small cell lung cancer, were included to ascertain the biosensors’ specificity. Alongside wells for biosensors interacting with target tumor markers, control wells were set up with biosensors reacting with bovine serum albumin (BSA) as a non-binding protein control and with NSE and proGRP. The fluorescence changes are reported as F/F0, with F0 being the basal fluorescence in PBS.

### 2.8. Detection of Clinical Sample

Clinical samples were collected from the Central Hospital of Dalian University of Technology from January 2024 to March 2024. Patients with tumors attending the Department of Thoracic Surgery were randomly enrolled, and serum samples before surgery were collected preoperatively with informed consent. Each serum sample to be tested was diluted 1:10 and tested in quintuplicate. A background control was set with serum control well, which contained 20 μL of serum and 180 μL of PBS, with the fluorescence value defined as F1. The fluorescence value of detection wells was defined as F2, which was the fluorescence value obtained by reaction of 20 μL of serum with 180 μL of biosensor. F0 represented the basal fluorescence of the biosensor in PBS. The fluorescence ratio was calculated using (F2 − F1)/F0, and the concentration of the target protein was determined from the standard curve.

### 2.9. Statistical Analysis

Data are presented as mean ± SD and were analyzed using Student’s t test or one-way ANOVA with Bonferroni post hoc test using GraphPad software (v9.0, GraphPad Software, San Diego, CA, USA). A *p*-value < 0.05 was considered statistically significant.

## 3. Results

### 3.1. Biopanning for Binding Peptides and Peptide Sequence Analysis

Phage display technology, which involves the fusing of exogenous proteins or peptides with phage coat proteins on the phage surface, was utilized to screen for specific protein or peptides through specific affinity interactions. A comprehensive biological screening procedure identified specific binding peptides, with the most specific binding peptides enriched after three rounds of screening. This enrichment was achieved by incrementally increasing the concentration of Tween 20 and the number of washing steps. Phage sequencing revealed 13 peptides specifically binding to CEA, 8 peptides to SCCAg, and 11 peptides to CYFRA 21-1 (Table 1).

**Figure 3:**
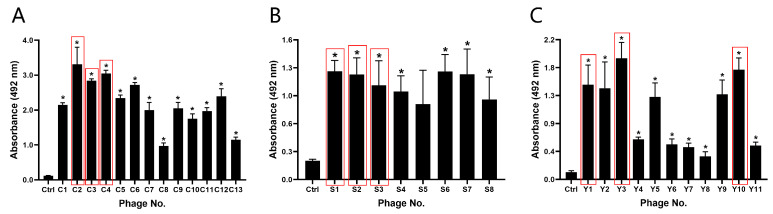
The binding affinity of the screened binding phages. Absorbance values measured at 450 nm from ELISA assays for CEA (**A**), SCCAg (**B**), and CYFRA 21-1 (**C**). The sequences in the red box were the phage sequences selected for each marker for subsequent experiments. * *p* ˂ 0.05 vs. Control group. (n = 3).

Phages selected based on sequence analysis were subjected to ELISA experiments to further verify the binding affinity between phages and the corresponding markers. The results demonstrated high affinity of the selected phages for their respective markers, confirming the effectiveness of the biopanning. Considering the number of repetitions in the screening stage and the affinity shown by ELISA, three sequences were selected for each marker for subsequent experiments, with C2, C3, and C4 for CEA (Figure 3A), S1, S2, and S3 for SCCAg (Figure 3B), and Y1, Y3, and Y10 for CYFRA 21-1 (Figure 3C).

### 3.2. Affinity Analysis of Screened Peptides

**Figure 4:**
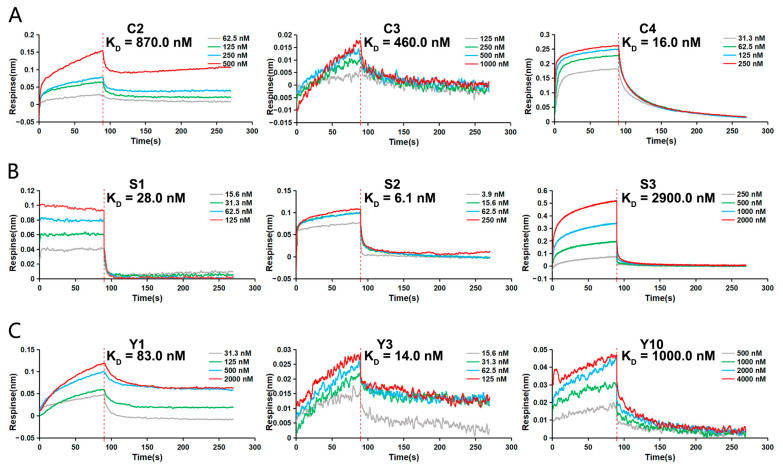
Affinity analysis of screened peptides. The binding affinities of the screened peptides to corresponding serum tumor markers was detected by BLI assay. (**A**) Determination of the K_D_ for the interaction between C2, C3, C4, and CEA, respectively. (**B**) Determination of the K_D_ for the interaction between S1, S2, S3, and SCCAg, respectively. (**C**) Determination of the K_D_ for the interaction between Y1, Y3, Y10, and CYFRA 21-1, respectively.

From the above experiments, three peptide sequences were obtained with high affinity for each marker. These peptides were synthesized to further evaluate the binding force by BLI detection. K_D_ serving as a measure of molecular interaction affinity in BLI indicates that a lower K_D_ value corresponds to a higher binding affinity. The BLI results demonstrated varying affinities among the peptides for their respective markers. For CEA, peptides C3 and C4 exhibited stronger binding with K_D_ values of 460.0 and 16.0 nM, respectively, compared to C2, which had a K_D_ value of 870.0 nM (Figure 4A). In the case of SCCAg, peptides S1 and S2 showed higher affinity with K_D_ values of 28.0 and 6.1 nM, respectively, than S3, which had a K_D_ value of 2900.0 nM (Figure 4B). For CYFRA 21-1, peptides Y1 and Y3 displayed stronger binding with K_D_ values of 83.0 and 14.0 nM, respectively, compared to Y10, with a K_D_ value of 1000.0 nM (Figure 4C). These data provide a quantitative assessment of the peptides’ binding affinities, identifying the most potent candidates for each marker.

### 3.3. Sensitivity Detection of the Biosensors

**Figure 5:**
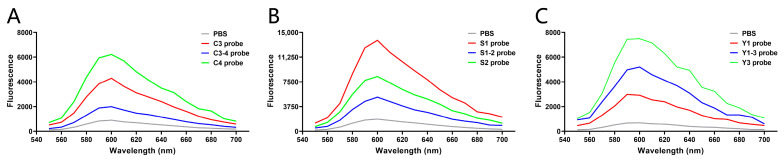
Sensitivity detection of the biosensors. Based on R-GECO1, the biosensors were constructed and synthesized according to the strategies, and the sensitivity of the reaction between each biosensor and corresponding tumor markers was detected. (**A**) Fluorescence values of PBS, C3 biosensor, C3-4 biosensor, and C4 biosensor mixed with CEA, respectively, tested by a microplate reader. (**B**) Fluorescence values of PBS, S1 biosensor, S1-2 biosensor, and S2 biosensor mixed with SCCAg, respectively, tested by a microplate reader. (**C**) Fluorescence values of PBS, Y1 biosensor, Y1-3 biosensor, and Y3 biosensor mixed with CYFRA 21-1, respectively, tested by a microplate reader.

Using R-GECO1 as the template, three cpFP biosensors were synthesized for each tumor marker according to the selected peptide sequence and two plasmid construction strategies, and the fluorescence changes of cpFP biosensors after reaction with the corresponding marker were detected. At the same concentration (4 μg/mL), the C4 biosensor (Figure 5A), constructed by linking two hexapeptides derived from C4 to the C-terminal and N-terminal of R-GECO1, showed the highest fluorescence value upon reaction with CEA. Similarly, among the biosensors designed for SCCAg and CYFRA 21-1, S1 (Figure 5B) and Y3 (Figure 5C) demonstrated the highest sensitivity to their respective markers. These findings indicate the effectiveness of the cpFP biosensors in detecting target tumor markers with high sensitivity.

### 3.4. Specificity Detection of the Biosensors

**Figure 6:**
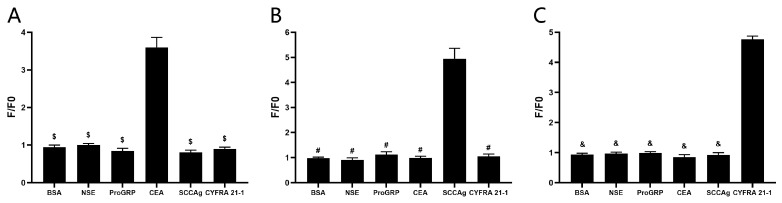
Specificity detection of the biosensors. (**A**) Changes in fluorescence of C4 biosensor upon reacting with BSA, NSE, proGRP, CEA, SCCAg, and CYFRA 21-1, respectively. $ *p* ˂ 0.05 vs. CEA group. (**B**) Changes in fluorescence of S1 biosensor upon reacting with BSA, NSE, proGRP, CEA, SCCAg, and CYFRA 21-1, respectively. # *p* ˂ 0.05 vs. SCCAg group. (**C**) Changes in fluorescence of Y3 biosensor upon reacting with BSA, NSE, proGRP, CEA, SCCAg, and CYFRA 21-1, respectively. & *p* ˂ 0.05 vs. CYFRA 21-1 group. (n = 5).

Based on prior experimental outcomes, the C4, S1, and Y3 were selected as cpFP biosensors of ECA, SCCAg, and CYFRA 21-1, respectively. To verify their specificity, a set of substrates including BSA, NSE, proGRP, CEA, SCCAg, and CYFRA 21-1 were reacted with the biosensors. As shown in Figure 6A, the fluorescence changes of the C4 biosensor reacting with CEA were significantly larger than those of C4 biosensor reacting with other tumor markers, while it can be seen in Figure 6B that the S1 biosensor showed significant fluorescence changes when reacting with SCCAg, but no significant fluorescence changes were observed when reacting with other tumor markers. Moreover, the Y3 biosensor targeting CYFRA 21-1 did not show significant fluorescence changes when reacting with other tumor markers but exhibited great fluorescence changes when reacting with CYFRA 21-1 (Figure 6C). Consequently, it has been inferred that the C4 biosensor (Figure 6A), S1 biosensor (Figure 6B), and Y3 biosensor (Figure 6C) exhibit commendable specificity.

### 3.5. Linearity Test for the Biosensors

**Figure 7:**
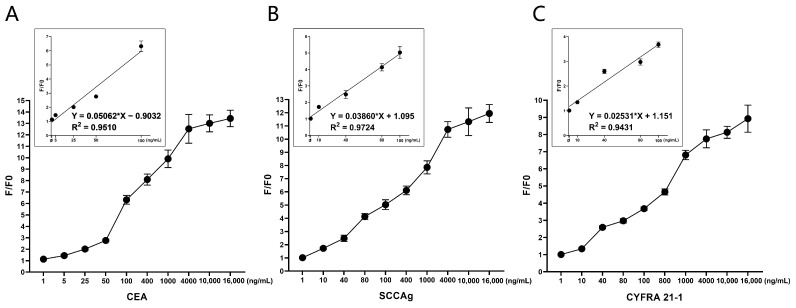
Linearity test for the biosensors. The reaction system consists of 180 μL/well biosensor (4 μg/mL) and 20 μL/well serum tumor marker. (**A**) Obvious fluorescence change was observed when C4 biosensor reacted with CEA, and F/F0 increased with the increase of concentration of CEA (1–16,000 ng/mL). The linear regression analysis of C4 biosensor was performed using 5 increasing concentrations of CEA: 1, 5, 25, 50, 100 ng/mL. (**B**) Obvious fluorescence change was observed when S1 biosensor reacted with SCCAg, and F/F0 increased with the increase of concentration of SCCAg (1–16,000 ng/mL). The linear regression analysis of S1 biosensor was performed using 5 increasing concentrations of SCCAg: 1, 10, 40, 80, 100 ng/mL. (**C**) Obvious fluorescence change was observed when Y3 biosensor reacted with CYFRA 21-1, and F/F0 increased with the increase of concentration of CYFRA 21-1 (1–16,000 ng/mL). The linear regression analysis of Y3 biosensor was performed using 5 increasing concentrations of CYFRA 21-1: 1, 10, 40, 80, 100. (n = 5).

At present, the quantitative assay kits (Toujing, Shanghai, China) were used at the Central Hospital of Dalian University of Technology for the detection of STMs. Their established upper limits of normal concentration for CEA, SCCAg, and CYFRA 21-1 are 5 ng/mL, 5 ng/mL, and 1.5 ng/mL [6], respectively. In this study, binding reactions were performed using 180 μL of biosensor solution (4 μg/mL) with 20 μL of STMs solution at different concentrations. Specifically, the C4 biosensor was reacted with CEA at 1, 5, 25, 50, 100, 400, 1000, 4000, 10,000, and 16,000 ng/mL (Figure 7A); the S1 biosensor with SCCAg at 1, 10, 40, 80, 100, 400, 1000, 4000, 10,000, and 16,000 ng/mL (Figure 7B); and the Y3 biosensor with CYFRA 21-1 at 1, 10, 40, 80, 100, 800, 1000, 4000, 10,000, and 16,000 ng/mL (Figure 7C). The experimental data revealed a progressive increase in the F/F0 value in correlation with the escalating concentrations of the protein standards. Considering the upper limits of normal concentration, the concentrations ranging from 1 to 100 ng/mL were selected for linear regression analysis. The results, as depicted in Figure 7, demonstrated that all biosensor curve fits had an R-squared (R2) value greater than 0.9, signifying a strong linear relationship. This indicates that the detection ranges of the biosensors are adequate to meet clinical requirements.

### 3.6. Evaluation of Assay Performance with Patient Samples

**Figure 8:**
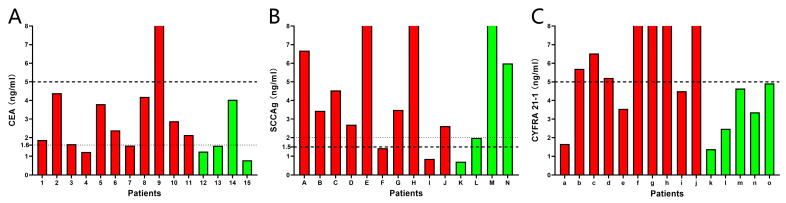
Evaluation of assay performance against patient samples. (**A**) The C4 biosensor was used to detect CEA in 11 NSCLC patients (red) and 4 non-NSCLC patients (green). (**B**) The S1 biosensor was used to detect SCCAg in 10 NSCLC patients (red) and 4 non-NSCLC patients (green). (**C**) The Y3 biosensor was used to detect CYFRA 21-1 in 10 NSCLC patients (red) and 5 non-NSCLC patients (green). The thick dashed lines represented the upper limit of normal concentrations currently used by the hospital. The thin dashed lines represented the adjusted upper limit of normal concentration for better diagnostic sensitivity and specificity.

A cohort of NSCLC and non-NSCLC patients including small cell lung cancer, lung benign space-occupying disease, pulmonary tuberculosis, and tumors in other locations, were randomly enrolled in the study. The results were analyzed based on the upper limits of normal concentration mentioned above. The C4 biosensor was used to detect serum samples from 15 patients, with 11 patients pathologically identified as NSCLC and 4 as non-NSCLC. According to the current clinic upper limit of normal concentration, only 1 out of 11 NSCLC patients has a CEA level higher than 5 ng/mL, while all non-NSCLC patients had CEA levels lower than 5 ng/mL (Figure 8A), indicating that this upper limit is not effective for aiding in the diagnosis of NSCLC. Therefore, the study adjusted the upper limit of normal CEA concentration to 1.6 ng/mL. With this adjustment, 9 out of 11 NSCLC patients and 1 out of 4 non-NSCLC patients were found to have CEA levels above 1.6 ng/mL. Although this adjustment led to a decrease in specificity, there was a significant enhancement in sensitivity, which is crucial for the early detection of NSCLC.

The S1 biosensor was utilized to analyze serum samples from 14 patients. Among 10 patients from NSCLC group, 8 patients had SCCAg concentrations higher than 1.5 ng/mL, while 3 out of 4 non-NSCLC patients had SCCAg levels above the same threshold (Figure 8B). By elevating the upper limit of the normal SCCAg concentration to 2.0 ng/mL, it was possible to reduce the false positive rate without affecting the positive detection rate.

The Y3 biosensor was applied to serum samples from 15 patients, and it was found that 7 out of 10 NSCLC patients had CYFRA 21-1 levels higher than 5 ng/mL, while all 5 non-NSCLC patients had CYFRA 21-1 levels lower than 5 ng/mL (Figure 8C). These results indicated that, based on the current upper limit of normal concentration, the Y3 biosensor demonstrated a sensitivity of 70% and a specificity of 100%.

## 4. Discussion

As genetically encoded fluorescent indicators, cpFP biosensors have risen as powerful tools for detecting life activities, owing to their high sensitivity and broad dynamic range. They have been widely utilized in the realms of biological imaging, drug screening, and cell metabolite detection [19]. To date, a variety of cpFP-based indicators has been developed for measuring inorganic ions such as Ca^2+^ [20] and Zn^2+^ [21], organic metabolites like glucose [22] and ATP [23], secondary messengers including DAG [24] and cGMP [25], neurotransmitters such as GABA [26], glutamate [27], and dopamine [28], kinase activity [29], and voltage changes [30], and even oxidation and reduction events [31]. However, the applications of cpFP for the detection of macromolecular proteins have been somewhat limited, potentially due to the requirement for antibodies as ligands to the target proteins. Although antibodies have high binding affinity and strong protein recognition specificity, their complex structure and high cost limit their application as binding domain proteins for cpFP-based biosensors. This study, therefore, introduces an innovative approach by employing peptides as binding domain proteins to construct cpFP biosensors.

Affinity peptides screened by phage display technology are characterized by their high affinity and selectivity, along with the advantages of simple and compact molecular structures, swift tissue permeability, rapid clearance, and minimal immunogenicity [32,33]. These peptides can be synthesized with modifications, serving as adaptive recognition elements for biosensors and thus enhancing their reliability, sensitivity, and selectivity. In addition, the synthesis of affinity peptides is highly reproducible and pure, facilitating cost-effective and rapid biosensor production [34]. In this study, CEA, SCCAg, and CYFRA 21-1 were used as ligands to screen peptides by phage display technology, and high-affinity peptides were selected through ELISA and BLI analysis and used as binding domain proteins to construct cpFP biosensors. Two strategies were employed for biosensor construction: one involved attaching two screened dodecapeptides to the N-terminus and C-terminus of cpFP, while the other divided the dodecapeptides into two hexapeptides to form binding domains at the termini. Sensitivity testing revealed that biosensors constructed using the latter strategy demonstrated heightened fluorescence responses upon interaction with their corresponding markers. As a result, this study successfully developed three effective biosensors, among which the C4 biosensor was used for CEA detection, the S1 biosensor for SCCAg and Y3 biosensor for CYFRA 21-1.

Following synthesis, the biosensors’ specificity was further verified, confirming their significant selectivity towards their respective STMs. The detection range was also explored, revealing that the biosensors have a broad detection range for their STMs and exhibit a strong linear response to the markers within the 1–100 ng/mL range. When applied to clinical samples, these biosensors demonstrated high sensitivity and specificity for NSCLC diagnosis, particularly after adjusting the upper limit of normal concentration. Compared to existing clinical methods, cpFP biosensors offer the advantage of low cost and ease of mass production through vector amplification and protein purification. Moreover, the biosensors enable rapid quantitative detection using a microplate reader, which is user-friendly and promotes ease of use. More importantly, the affinity peptides exhibit high selectivity for target proteins, ensuring that biosensors constructed with these affinity peptides as binding domains exhibit robust specificity, fluorescence response, and sensitivity, which are crucial for the diagnosis of NSCLC and hold broad application prospects.

While the BLI results did not show a significant advantage in binding affinity between affinity peptides and target proteins compared to antibodies, the biosensors still exhibited high fluorescence responses. We believe that enhancing binding affinity through mutation or further biopanning could potentially improve the sensitivity and specificity of the biosensors. To achieve appropriate diagnostic sensitivity and specificity, it is essential to expand the sample size and more accurately determine the upper limits of normal concentrations for the biosensors. Additionally, these cpFP biosensors, constructed using affinity peptides as binding domain proteins, exhibit good tissue compatibility and may enable in vivo visualization and boundary determination of lung cancer, which warrants further investigation.

## 5. Conclusions

This study successfully leveraged the combined strengths of peptides and cpFP to engineer novel biosensors for the detection of CEA, SCCAg, and CYFRA 21-1. These biosensors demonstrated high sensitivity and specificity for detecting target proteins and diagnosing NSCLC, and the detection process was simple and easy to operate. In addition, the low-cost was suitable for mass production of these biosensors position them as valuable tools in the screening and early diagnosis of NSCLC.

## Figures and Tables

**Table 1:** Peptide sequence of the selected binding phage.

CEA			SCCAg			CYFRA 21-1		
No.	Peptide Sequence	Repetition	No.	Peptide Sequence	Repetition	No.	Peptide Sequence	Repetition
C1	VVGRAMAYSTIP	7	S1	TLSWHQNLRLME	3	Y1	CFAGTPSILMLA	18
C2	YGVSALSSYVSC	4	S2	AYSRDVVLNMWR	2	Y2	GVGNFAPYWHMV	3
C3	WSPSALLPSSVT	2	S3	IQCFSLAPYVGC	2	Y3	GMCSFVDVANCP	3
C4	HHRLTRSMQLMM	2	S4	NAPQASQVWKGL	1	Y4	VVGRAMAYSTIP	3
C5	FQAPYWLTLGGE	1	S5	LNVTNSVYPGIR	1	Y5	DFSPRGSSISPF	2
C6	DVSPSRNQDRSP	1	S6	SVYNALYLAASE	16	Y6	AIVPFQMWERIQ	1
C7	VNPFHKFTAGNQ	1	S7	VVGRAMAYSTIP	1	Y7	NTNGFHKYHLSR	1
C8	ALNGVKGPLRMD	1	S8	MDREAHRMVQAT	1	Y8	SPIVQQRPVTGK	1
C9	FPRLTALAGPWP	1				Y9	HYTADTPHRWPL	1
C10	DVTWRTSYSSDS	1				Y10	QSYFNACWSCNH	1
C11	WSLNSGMFGYQW	1				Y11	AKNSDYKMWVLG	1
C12	DRGSGVPADELW	1						
C13	SWFQSDNTLRRP	1						

## Data Availability

The raw data supporting the conclusions of this article, which are not publicly available, will be made available by the authors, without undue reservation.
